# Identifying confounders and estimating the causal effect of antenatal care on age-specific childhood vaccination

**DOI:** 10.3389/fpubh.2025.1420567

**Published:** 2025-05-30

**Authors:** Ashagrie Sharew Iyassu, Haile Mekonnen Fenta, Zelalem G. Dessie, Temesgen T. Zewotir

**Affiliations:** 1 Department of Statistics, College of Science, Bahir Dar University, Bahir Dar, Ethiopia; 2 Department of Statistics, Debre Markos University, Debre Markos, Ethiopia; 3 Population Health, Center for Environmental and Respiratory Health Research, University of Oulu, Oulu, Finland; 4 School of Mathematics, Statistics and Computer Science, University of KwaZulu-Natal, Durban, South Africa

**Keywords:** antenatal care, childhood immunization, confounders, significance testing, change in estimate

## Abstract

**Background:**

Immunization is an efficient and cost-effective public health program. It averts millions of child deaths per year. It is taken as one of the main interventions that can be used to achieve the third Sustainable Development Goal, which is to end preventable deaths of newborns and under-five children by 2030. The study was done with the aim of identifying appropriate confounder identification methods and examining confounders for the causal effect of a number of antenatal care visits on age-specific childhood vaccination.

**Methods:**

A family of generalized linear models with log link functions was used to model the covariate and the number of antenatal care association. A cumulative link model was used to model the number of antenatal care and covariate-age-specific childhood vaccination associations. AIC and BIC values were used to compare models. Significance testing methods and change in estimate methods were used to identify covariates that confound the effect of a number of antenatal care on age-specific childhood vaccinations.

**Result:**

A zero-inflated Poisson model was selected to model covariate–exposure association, and a proportional odds model with a log link was selected to model the outcome variable. Among significance testing methods, the common cause approach yielded smaller values of BIC and a smaller number of covariates. However, the likelihood ratio test showed no difference between the common cause and other approaches. A change in the estimate method is more conservative at a 10% cut point, which selects a smaller number of confounders. However, the significance testing method was better performed than the change in estimate method.

**Conclusion:**

The significance testing method with a *p*-value of less than or equal to 0.2 performed better than a change in estimate method at a 10% cut point of effect change for confounder identification. Mothers’ age at first birth, region, place of residence, education status of mothers, presence of radio and television in the household, religion, household size, wealth status, total children ever born, and birth order number are identified as confounders.

## 1 Introduction

Most newborn and under-five deaths in sub-Saharan African countries are caused by childhood diseases that can be prevented through immunization. Immunization is an efficient and cost-effective public health program that averts approximately 2.5 million child deaths in a year and is considered to be one of the principal interventions that can be used to accomplish the third Sustainable Development Goal (SDG), which is to end preventable mortality of neonatals and under-five children by 2030 (1–3).

The World Health Organization (WHO) introduced the Expanded Program on Immunization in 1974. The program recommends that immunization should be 90% at the state level and at least 80% at the district or equivalent administrative level for children aged 1 year (4).

Ethiopia launched its Expanded Program on Immunization in 1980 with vaccines of Bacillus Calmette–Guerin (BCG), Diphtheria, pertussis, tetanus, polio, and measles. Later, in 1986, the program was revised with a target of 75% coverage, and the target age group was infants less than 1 year old. However, the progress has been slow in increasing immunization coverage. After the introduction of a new approach in 2003, known as reaching every district and sustainable outreach for immunization, improvement has been recognized (5).

A child who is aged 12–23 months has received all the immunizations recommended by the extended immunization program is considered to have received a full vaccination (6–10). However, for the effectiveness of vaccination, timing is very important. A timely start to vaccination is critical in the first year of life as transplacental immunity decreases fast, and timely administration of vaccination has consequences for the efficacy of pediatric immunization programs (11). Early or late administration of vaccination reduces the impact of vaccine programs on disease burden, especially in high-risk groups (12).

For example, except for BCG and polio at birth, any vaccine administered before 6 months has shown poor response and, in some cases, could be harmful to infants as they reduce the immune response of subsequent doses. Hence, administering vaccines before schedule or closer to each other may lead to a suboptimal immune response. Conversely, the optimal level of vaccine protection may not be achieved if a child’s vaccination is delayed and the time between doses/vaccines is lengthened (13). Both individual and herd immunity are compromised when vaccines are given with considerable delays, which is not surprising given that outbreaks of diseases such as pertussis or measles will happen (14).

In an observational study, confounders have to be identified and their effect controlled while estimating the association between exposure and outcome. Controlling confounders helps to obtain unbiased estimates of the exposure–outcome relationship (15). Including all pre-treatment covariates in any confounder controlling methods, such as regression, introduces bias. Adding more covariates to the model causes over-fitting and unstable coefficients due to multicollinearity (16). A model is best when it contains the smallest number of covariates that explain the greatest amount of variance (17). As a result, identifying confounders that potentially distort the causal effect of treatment on the outcome is imperative. However, identifying confounders and dealing with them is one of the challenges in observational studies. There is no common consensus criterion for identifying which covariates are confounders and which are not (17). A common approach is to control for as many pre-exposure covariates as possible (18). Some studies have modified this approach by controlling all covariates that are significantly associated (*p*-value less than 0.05) with the outcome of interest (19, 20), as mentioned in Ref. (18). Others have stated that control confounders provide a predetermined magnitude of change, typically 10% or 15%, in estimating the relationship between exposure and outcome (19, 21).

Confounders that distort the causal effect of antenatal care on age-specific or timely vaccination have not been documented yet. In addition, little research has been conducted on comparing confounder identification techniques, particularly with count exposure such as frequency of mothers’ antenatal care services (ANCs) at health facilities. Accordingly, identifying and controlling for confounders will be crucial in determining the true relationship between the frequency of antenatal care and age-specific childhood immunization. Hence, the aim of this study was to determine the best confounder identification technique, to determine confounders that affect the causal effect of ANC on age-specific childhood vaccination, and to estimate the effect of ANC on age-specific childhood vaccination after adjusting for confounders with the regression method.

## 2 Data and methods

### 2.1 Source and description of data

Data were obtained from the Ethiopian Mini Demographic and Health Survey (EMDHS) collected from 21 March 2019 to 28 June 2019. Data obtained from birth records included all records of women aged 15–49 years with the most recent birth within 5 years prior to the survey. In the survey, 5,753 women with live births were interviewed (22). However, only children who were alive at the time of the survey were considered for this study, which is because we could not find the vaccination history of deceased children in the dataset.

### 2.2 Description of variables

A child is considered fully vaccinated when receiving BCG and OPV0 at birth; DTP-HepB1-Hib1, OPV1, PCV1, and Rota1 at 6 weeks of birth; DTP-HepB2-Hib2, OPV2, PCV2, and Rota2 at 10 weeks of birth; DTP-HepB3-Hb3, OPV3, PCV3, and IPV at 14 weeks of birth; Measles at 9 months of birth; and vitamin A supplement until 59 months of birth (5). When a child received a particular vaccination, a score of 1 was given; otherwise, 0 was given. With these scores, a composite index was calculated. When a child received all vaccines on time, it was labeled as fully vaccinated. If one or more vaccines were missed at each age, the child was labeled as partially vaccinated and labeled as not vaccinated when a child took no vaccination at each age.

**Figure 1:**
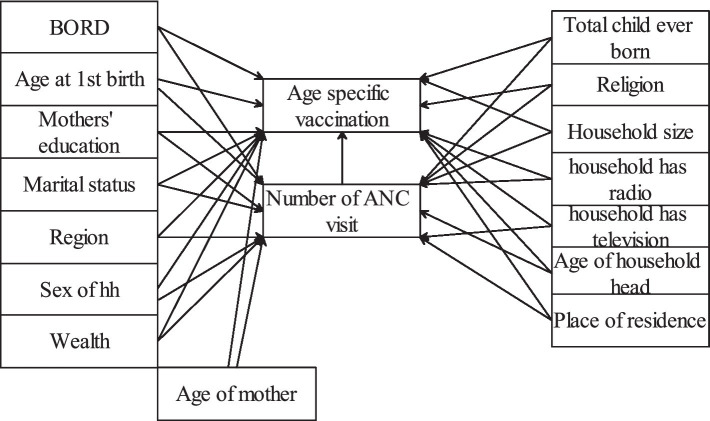
Conceptual framework.

We considered antenatal care as the causal/treatment variable that causes the age-specific childhood vaccination status. The conceptual framework illustrating the relationship between pre-treatment covariates or possible confounders, exposure, and outcome is shown in Figure 1. These pre-treatment covariates were selected from a literature review. The availability of covariates in the dataset was checked before adding them into the framework.

The exposure and outcome variables had missing observations. Missingness can be classified as missing completely at random (MCAR), missing at random (MAR), and missing not at random (MNAR) (23). MAR is a more relaxed assumption where the probability of missingness is dependent on the observed covariates but independent of the unobserved covariates (24). When missing data are MAR, a valid conclusion can be drawn using appropriate imputation techniques such as multiple imputation (25). In contrast, analysis under MNAR is more challenging since some relevant information remain unobserved and additional, untestable assumptions are required to proceed with the analysis. As a result, the MAR assumption is a commonly used starting point in missing data analysis (26). In this study, we focused on missing at random (MAR).

**Figure 2:**
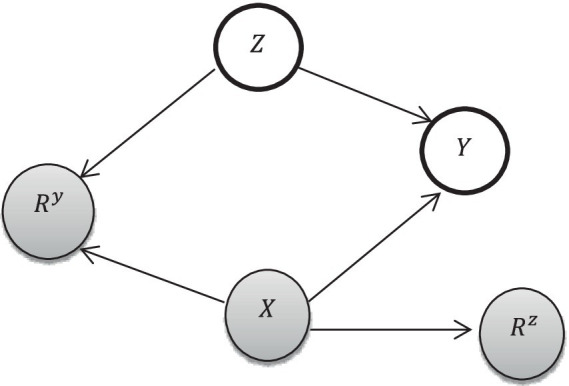
Missingness conceptual framework [adapted from Ji et al. (27)].

Let Z be the number of antenatal care visits (exposure variable),X be covariates, and Y be the childhood vaccination status (outcome variable).X is observed, and Z and Y are missing, which have two components. $R^{z}\;\text{and}\;R^{y}$ are missingness indicators for ZandY,respectively, where a value of 1 indicates that ZandY are missing and 0 indicates that the variables are observed. The missing graph (DAG), also called “m-graphs” (27) for the MAR assumption, is presented as follows. The whole circle indicates missing, and the shaded circles indicate observed (Figure 2).

### 2.3 Missing data management

Missingness for vaccination ranges from 41.03% for BCG to 42.46% for vitamin A supplementation. The overall missingness of childhood vaccination was 48.8% (see Supplementary material for all missing distributions). While this appears to be a high level of missingness, Graham (28) stated that multiple imputations work very well, even with 50% missing of the dependent variable. The absolute bias and MSE were smaller under all missing mechanisms for a high percentage of missingness, even up to 80% missingness (29). Similarly, Faria R., et al. (30) used multiple imputations for 51% of missingness, and White et al. (31) used imputations for 78% of missingness. For antenatal care, 29.87% of observations were missing. The missing data were not available in the dataset but would be meaningful if they had been available. To protect against loss of information due to complete case analysis (assuming missing completely at random), a test of missing at random (MAR) was conducted using the regression method as follows:

First, a column with missing values (childhood vaccination and number of antenatal care) was dichotomized into 0 and 1 such that $R=\{\begin{array}{c} 0\;\text{if observation is observed}\\ 1\;\text{if observation is missed}\; \end{array}$

Second, the dichotomized variable was regressed against the observed covariates using logistic regression (32). First, a bivariate analysis was conducted, followed by a multivariable logistic model using variables with a significant value of (*p* of <0.2 in the bivariate analysis).

As a result of missingness being associated with observed values, multiple imputation was used to obtain consistent, asymptotically efficient, and asymptotically normal estimates (33). The ordinal model for childhood vaccination status and the Poisson model for a number of antenatal care visits were used for imputation using observed significant covariates. The multiple imputation was conducted using the *mi impute* (34) Stata command. We created 50 and 30 datasets for outcome and exposure variables as per the rule of thumb by White et al. (31), which suggests that the number of imputed datasets should be at least as large as the percentage of missing data. The number of imputations should be at least the proportion of missing data (30). As a result of multiple imputations, we conducted a sensitivity analysis on the effect of the exposure on the outcome before and after imputation, such that the result is given in Supplementary material. The imputation increased data values from 3,208 to 5,150, and the coefficient of the exposure decreased from 0.576 (based on complete case analysis) to 0.17 (based on multiple imputation). The width of confidence interval decreased from 0.164 to 0.032. These results suggest that multiple imputation yielded more precise estimates than the complete case analysis.

### 2.4 Methods of identifying confounders

Adjusting for a set of covariates assumed to be confounders, those that are capable of producing spurious associations between exposure and outcome, is the common method of estimating causal effects in observational studies (35). For a variable to be confounder, it should not be in the causal pathway between exposure and outcome, and it must be unequally distributed between study subjects (36, 37). An important step before applying statistical methods to correct confounders is identifying covariates that confound the causal effect of a number of antenatal care interventions on age-specific childhood vaccinations. The methods used to identify confounders included significance testing based on *p*-values and change in estimate.

Statistical testing or the *p*-value method was applied after identifying data-driven statistical models for the relations between cofounder and exposure and between cofounder and outcome. A threshold *p*-value of 0.2 was used to identify a covariate as a cofounder (15, 38).

In this case, three approaches were compared. The first approach involved selecting common covariates that were significant for both exposure and outcome. The second approach involved selecting covariates that were significant for either the exposure, the outcome, or both. The third approach treated all pretreatment variables as confounders. To compare the performance of each approach, AIC, BIC, and likelihood ratio tests were used after regressing the selected covariates and exposure on the outcome variables. The significance of covariates was evaluated based on a threshold value of *p* of 0.2 during the bivariate analysis. Moreover, the relative change of exposures’ (ANC service) effect on outcome (childhood vaccination status) was taken into account to select the approach.

The change in estimate is a method of identifying confounders based on the inclusion of covariate changes in the estimate of the causal effect of exposure on the outcome (39) by more than the specified threshold value, typically 10% (40).

In divergence with significance testing, the change-in-estimate (CIE) approach identifies covariates based on how much their control changes exposure’s effect estimates, regardless of significance or *p*-value; the observed change is supposed to measure confounding by the covariate (41). We have used two approaches to measure the change in the estimate, which are the CIE based on the coefficient and based on the attributable fraction (AF) using the odds ratio (41). Let $\beta_{a}$ be the coefficient of exposure on outcome without a covariate, Z and $\beta_{z}$ be the coefficient of exposure on outcome when a covariate, and Z is added to the exposure-outcome relationship, then the relative change in the estimate due to the covariate Z is estimated as $\Delta =|(\beta_{a}-\beta_{z})/\beta_{a}|.$ In applying change in estimate (CIE), the covariate Z is considered cofounder and included in the final model when Δ>0.1or10% (15).

When taking into account the odds ratio, let ORa and ORu denote the estimated odds ratio with and without the adjustment of covariates; then, RRa/RRu is the conventional method of measuring change in estimate or change in importance. However, Greenland S and Pearce N (41) suggested that the attributable fraction (AF)=(OR−1)/OR is more relevant and change in estimate could be measured by |AFa-AFu|.

A comparison of change in estimate effect and significance testing methods of confounder identification is done using the likelihood ratio test, Akaike information criterion (AIC), Bayesian information criterion (BIC) values, and changes in exposure’s effect on outcome.

### 2.5 Statistical models

When significance criteria and change in estimate methods are used for cofounder identification, the true causal relationship between the exposure and the outcome, as well as the set of confounders, remains unknown (15). Hence, it is important to propose data-driven statistical models for a better explanation of such a relationship.

The exposure variable in this study, i.e., the number of antenatal care of pregnant women with values ranging from 0 to 11, is the count variable. Hence, a family of count models is used to model the relationship between pre-exposure covariates and exposure. The models can be categorized into two broad families: the generalized linear model (GLM) family with a log link and the zero-augmented family. The GLM family includes Poisson regression and its extension, that is, negative binomial regression. The zero-augmented family includes zero-inflation Poisson and zero-inflated negative binomial regression (42).

Taking $y_{i},i=0,1,2,\ldots ,11$ and vector of covariates, the probability density function for the generalized linear model is $f(y,\gamma ,\emptyset )=\exp(\frac{y.\gamma -b(\gamma )}{\emptyset }+c(y,\emptyset ))$, where γ is a canonical parameter and ∅ is a dispersion parameter. The Poisson GLM is g(E(y))=x′β with canonical link function of g(E(y))=logE(y). The mean and variance are equal E(y)=var(y)=μ, and the dispersion parameter ∅ is 1. However, when there is overdispersion, ∅>1, the Poisson GLM is negative binomial with the same canonical link function (42).

For the outcome variable, one set of observations may be necessarily zero and the other set may be zero due to a random event, which naturally points to a mixture model in which two types of zeros can occur. The relevant distribution is a mixture of an ordinary count model, such as the Poisson or negative binomial, with one that places all its mass at zero.

According to Lambert (50), mentioned in (43), the zero-inflated model assumes


$$y_{i}\sim \{\begin{array}{c} 0\;\text{with probability}\;1-\theta_{i}\\ \text{poison}(\gamma )\;\text{with probability}\;\theta_{i} \end{array}$$

The unconditional probability is given by $P(y_{i}=0)=(1-\theta_{i})+\theta_{i}e^{\gamma_{i}}$ and $P(y_{i}=j)=\theta_{i}\frac{e^{\gamma_{i}}}{j!}{\gamma_{i}}^{j}$.

Considering a vector of covariates, the zero-inflated model, which is a mixture of two models, is given by logit(*θ*$)=x\prime_{1}\beta_{1}$ and $\;\log(\gamma )=x\prime_{2}\beta_{2}$. $x_{1}$ and $x_{2}$ may or may not be the same. In case of over-dispersion, $y_{i}$ is negative binomial with mean γ and dispersion parameter ∅ (43).

On the odified this approach by con-specific childhood vaccination) is ordinal, as no vaccination is coded as 0, partial vaccination is coded as 1, and full vaccination is coded as 2. The relationship between exposure and outcome, as well as covariates, is modeled with a proportional odds or commutative link model. Let $y_{j}$, j=0,1,2 be the status of age-specific childhood vaccination, z be the exposure variable, and x be the vector of covariates, then the cumulative link model is given by: $\text{logit}(P(y\leq j)=\log(\frac{P(y\leq j)}{1-P(y\leq j)})=\alpha_{j}-(\theta *Z+x\prime \beta )$. The assumption of the cumulative link model is that, except for the intercept, the effect of the covariate and exposure is constant for each increase in the level of the response. If this assumption fails to hold, a partial proportional model (a mixture of ordinal and multinomial models) is used.

Furthermore, different link functions of the proportional odds model were compared. The analysis was conducted using the R ordinal package (44). The Akaike information criterion (AIC) and Bayesian information criterion (BIC) (45) were used to compare and select appropriate count models.

## 3 Result and discussion

### 3.1 Results

#### 3.1.1 Descriptive analysis

**Figure 3:**
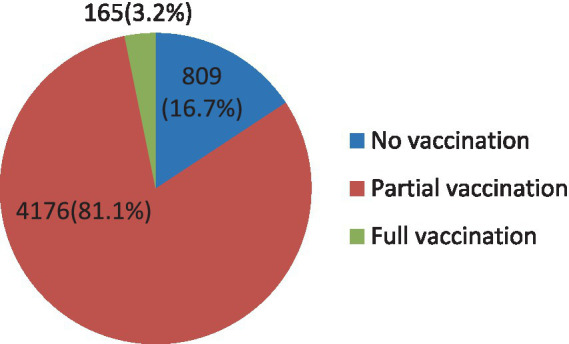
Age-specific vaccination status.

The result from the EDHS 2019 survey revealed that age-specific vaccination was very low (Figure 3). Only 3.2% of children were administered full vaccination at the right age, whereas the largest proportion of children (81.1%) took at least one but not all immunizations at the right time. On the other hand, a considerable number of children (16.7%) did not receive any vaccination at the right age.

**Figure 4:**
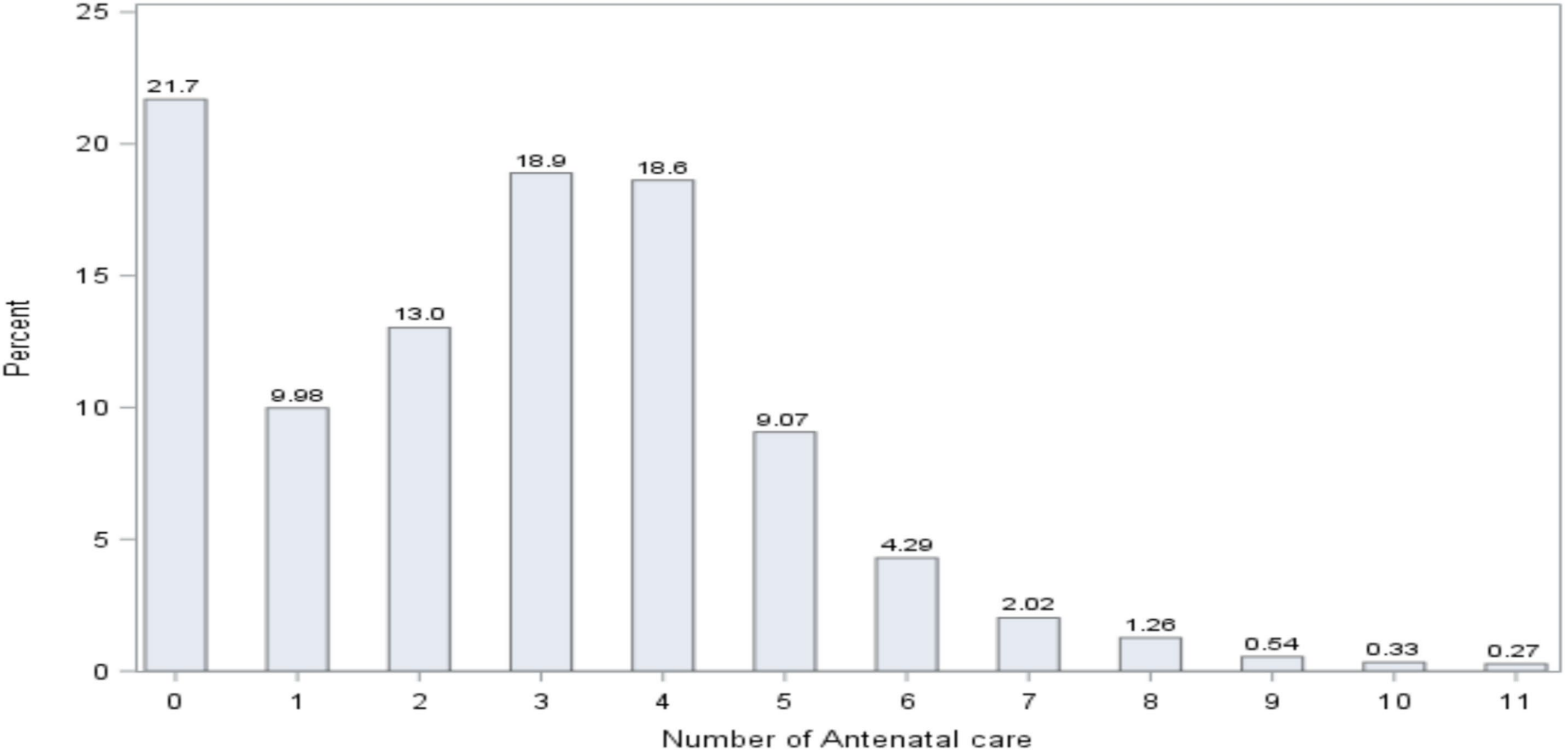
Distribution of the number of antenatal care.

The distribution of the number of women in antenatal care (ANC) is presented in Figure 4. The figure demonstrates that 21.7% of women did not follow any ANC; 9.98% of women followed 1 ANC; 13% of women followed 2 ANC; 18.9% of women followed 3 ANC; and 18.6% of women followed 4 ANC.

The result further indicated that 41.88% of women did 1–3 ANC visits, and a smaller number of women (36.38%) received WHO’s recommended number of ANC visits (4 + ANC visits) (46).

**Figure 5:**
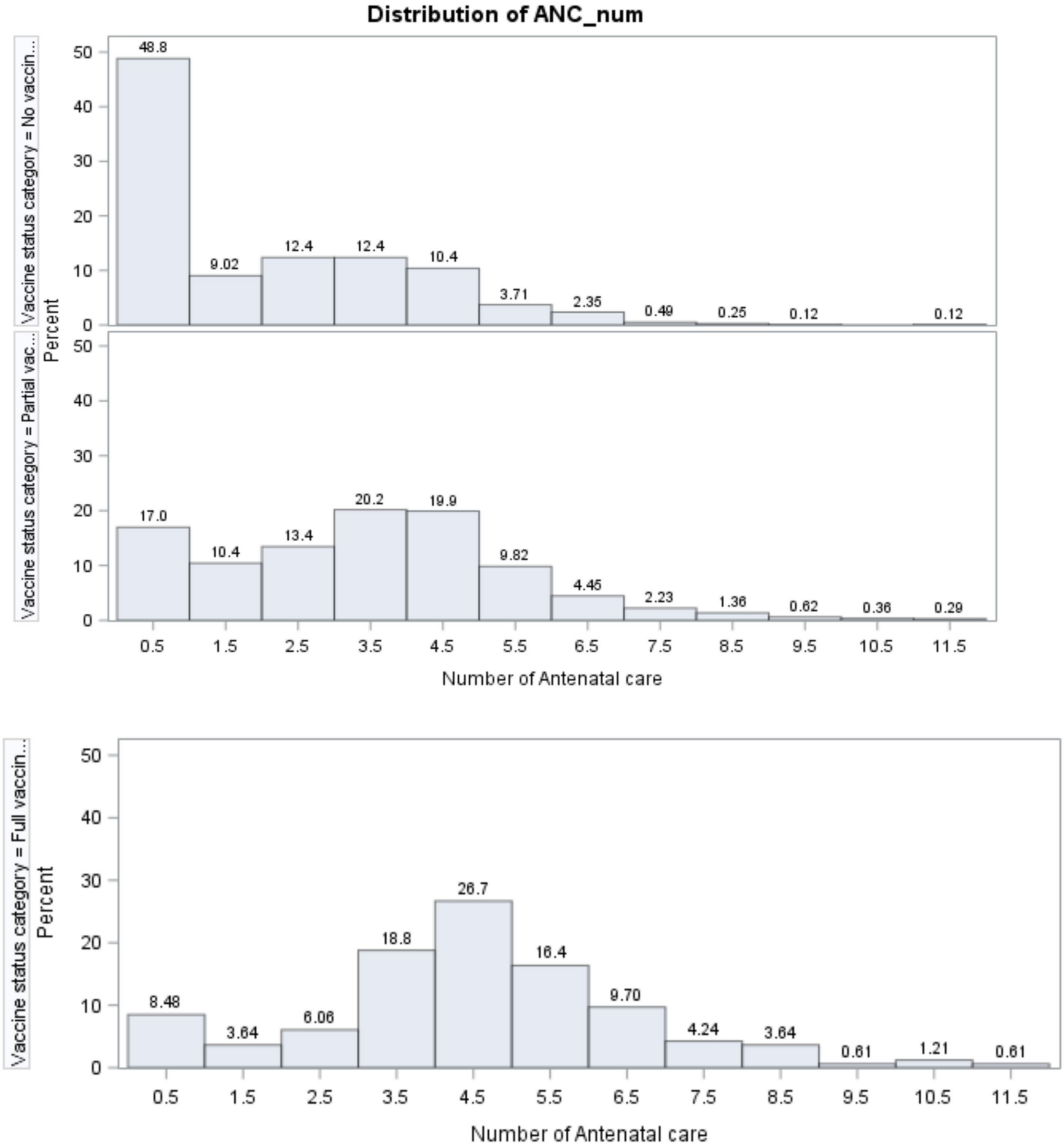
Distribution of the number of ANC across age-specific childhood vaccination.

The distribution of the number of ANC visits for each age-specific childhood vaccination status is presented in Figure 5. Among non-vaccinated children, the majority of pregnant women (48.8%) did not attend any ANC checkups. For those with three and four ANC visits, 12.4% of women participated in each category, and this number dropped to 0.12% for women with 10 and 11 ANC visits.

For partially vaccinated children, the number of women with no ANC (17%) was less than that of women (48.8%) with no childhood vaccination. Relatively, the majority of pregnant women (20.2 and 19.9%, respectively) visited health facilities for ANC checkups four and five times, which was a large number compared to no age-specific childhood vaccination.

Figure 5 further implied that, for full age-specific childhood vaccination, the number of pregnant women with no ANC visit (8.48%) was smaller than the number of women with no vaccinated children (48.8%) and the number of those with partially vaccinated children (17%). Similarly, 26.7% of women visited health facilities five times for ANC checkups. This number is greater than the number of women with no childhood vaccination and those with partial age-specific childhood vaccination. From this result, it can be inferred that the number of ANC visits by women is associated with the status of age-specific childhood vaccination.

#### 3.1.2 Model selection for covariate–exposure relationship

The Log-likelihood, BIC, AIC values for candidate models are summarized in Table 1. The result revealed that the zero-inflated Poisson regression model had smaller deviance (18439.54), AIC (18611.54), and BIC (19174.56) values as compared to the negative binomial model (deviance = 18446.83, AIC = 18620.83, and BIC = 19190.40). Hence, the test values of zero-inflated Poisson models were smaller than the other three candidate models (Table 1) and selected for confounder identification, especially for the significance testing approach.

**Table 1:** Model comparisons.

Model	-2*Log likelihood	AIC	BIC
Poisson	19984.44	20068.44	20343.40
Negative binomial	19924.87	20010.87	20292.38
Zero-inflated poisson regression	18439.54	18611.54	19174.56
Zero-inflated negative binomial	18446.83	18620.83	19190.40

#### 3.1.3 Model selection for covariate–outcome relationship

From Table 2, one can observe that the AIC and BIC values of the logit link were 5,198.17 and 5,486.22, respectively. For the complementary log–log (Cloglog) link function, the AIC and BIC values were 5,536.52 and 5,824.58, respectively. The result further demonstrated that the AIC and BIC values of the log–log link function were 5,105.11 and 5,393.17, respectively. For the cauchit link, the AIC and BIC values were 5,136.43 and 5,424.49, respectively. However, for Aranda-Ordaz and log-gamma links, the analysis did not converge. By observing the AIC and BIC values in Table 2, the model with the log–log link function was found to have the smallest values and to be the optimal model.

**Table 2:** Model comparison for the outcome variable.

Link functions for cumulative link model	AIC	BIC	Remark
logit	5198.17	5486.22	
Cloglog	5536.52	5824.58	
Log–log	5105.11	5393.17	
Cauchit	5136.43	5424.49	
Aranda-Ordaz			Does not converge
log-gamma			Does not converge

#### 3.1.4 Confounder identification using significance testing

The result in Table 3 shows that the BIC value of approach 1 was smaller than that of other approaches, despite the small difference. The number of covariates in approach 1 was smaller than that in the other two approaches. However, the likelihood ratio test was insignificant in all approaches.

**Table 3:** Comparing approaches to confounder identification.

Approaches	AIC	BIC	Likelihood ratio test	
			LR.statistics	*p*-value
Approach 1: common cause	5013.65	5262.42		
Approach 2: Cause for treatment or outcome or both	5013.04	5268.36	2.6094	0.106232
Approach 3:Pretreatment	5021.55	5316.16	3.4837	0.746142

Thus, covariates significant in both exposure and outcomes were identified as confounders for a causal effect of exposure on the outcome due to the smaller value of BIC and a smaller number of confounders than others. Accordingly, the result in Supplementary material showed that mothers’ age at first birth, region, place of residence, education status of mothers, presence of radio and television in the household, religion, household size, wealth status, total children ever born, and birth order number were identified as confounders.

On the other hand, the CIE for the effect of antenatal care on age-specific childhood vaccination, before and after controlling for confounders for all approaches of the significance testing method, is shown in Table 4. The result demonstrated that the linear change of ANC on the log–log of cumulative probability did not vary significantly in all approaches. Similar results were observed in the change of the odds ratio for ANC across all approaches. When we compared the three approaches, nearly all exhibited the same change of estimate for the effect of the number of antenatal care on the outcome, i.e., age-specific childhood vaccination.

**Table 4:** Change of ANC effect on age-specific childhood vaccination.

Approach	Coefficient	Odds ratio	Linear change of coefficients	Change of odds ratio
Unadjusted ANC	0.160723	1.17436		
Approach 1: Common cause of treatment or outcome covariates	0.102770	1.108236	0.057953	1.0597
Approach 2: Cause of treatment or outcome or both covariates	0.102752	1.108217	0.057971	1.0597
Approach 3: All pre-treatment covariates	0.102638	1.10809	0.058085	1.0598

Similar to the result in Table 3, we can choose a common cause as a confounder identification approach when using a significance testing approach.

#### 3.1.5 Confounder identification using change in estimate (CIE)

Table 5 presents a change in estimate of an exposure when covariates are included in the exposure-outcome model. Using threshold values of 9% for CIE based on coefficients or 1 for CIE based on AF, region, place of residence, education status, existence of television at home, and household wealth status were identified as confounders that alter the effect of the exposure (number of antenatal care at pregnancy) on the outcome (age-specific childhood vaccination).

**Table 5:** Result of the change in estimate.

Exposure	Coefficient of ANC	CIE (coeff.)	CIE (AF)
ANC alone	0.16072		
Age of mothers	0.16403	2%	0.281391
Region	0.12812	20%	2.82173
place residence	0.1421	12%	1.60045
Education status	0.14704	9%	1.17294
Radio	0.15664	3%	0.34818
Television	0.14425	10%	1.41413
Religion	0.14927	7%	0.98065
Household size	0.16089	0%	0.014428
Sex of household head	0.16068	0%	0.00345
Age of household head	0.16119	0%	0.039966
Household wealth status	0.1229	24%	3.28221
Total children ever born	0.16164	1%	0.078258
Age at 1st birth	0.159	1%	0.14664
Marriage status	0.16109	0%	0.031454
Birth order number	0.15852	1%	−0.18759

#### 3.1.6 Comparison of significance testing and change in estimate

The result in Table 6 shows that the likelihood ratio test favors the significance testing method of confounder identification. The AIC and BIC values of significance testing were smaller than that of the change in estimate.

**Table 6:** Comparison of methods.

Confounder selection technique	Number of covariates	AIC value	BIC value	Likelihood ratio test	
				LR.stat	Pr(>Chisq)
Change in estimate	5	5282.7	5426.699		
Significance testing	11	5075.8	5285.313	226.85	< 2.2e-16

Considering significance testing as a better approach to confounder identification, mothers’ age at first birth, region, place of residence, mothers’ education status, having radio and television, religion, household size, household wealth status, total children ever born, and birth order number were identified as confounders for the causal effect of the number of the antenatal care service on age-specific childhood vaccination (the result is presented in Supplementary material).

#### 3.1.7 Estimating the effect of ANC on age-specific childhood vaccination

The result of estimating the causal effect of ANC on age-specific childhood vaccination while controlling for identified confounders using a cumulative link model is provided in Supplementary material. It shows that the coefficient of ANC on the cumulative link model was 0.101, which indicated that the ANC follow-up had a positive and significant effect on the probability of higher-order categories of age-specific childhood vaccination status. When a woman visits a health facility for ANC services, the probability that her newborn baby will get the vaccination at the right age increases.

## 4 Discussion

The purpose of identifying confounders is to obtain minimally sufficient covariates and control them using statistical methods such as regression and estimate the association between exposure and outcome (47). On the other hand, including all pre-treatment covariates in the regression model to adjust them causes overfitting since some covariates retained in the model may be noisy, in that the model will not be reproducible for other datasets (16). Two principal methods were explored for cofounder identification, which were significance testing and change in estimate methods.

Count models were compared concerning their performance in relation to the relationship between pre-treatment covariates and the treatment. Among others, zero-inflated Poisson regression was found to be the best fit. Furthermore, a cumulative link or proportional odds model with various link functions was proposed for pre-treatment covariates and outcome variables. Accordingly, log–log was found to be the best fit and used in significance testing and change in estimate methods.

Selecting all pre-treatment covariates as confounders is one approach to confounder identification. In this approach, all covariates before the exposure should be controlled in causal inference (39). The “common cause” approach of confounder identification is controlling all covariates that are significantly associated with the exposure and the outcome (48). The other approach, which is intermediate between the two, is controlling confounders that are significant causes of the exposure or the outcome or both (39). In this study, all three approaches were tested using a significant testing method of cofounder identification. The likelihood ratio test shows that there is no difference in which method to use. Similarly, the change in the log-odds ratio of the coefficient of ANC is almost similar in common cause and a cause of either the treatment or the outcome or both. However, considering and adjusting all pre-treatment covariates provides a relatively small change as compared to the other two approaches.

The CIE is efficient when the cut-off point is set to 10%, with and without adjustment of covariates (38). For this study, a cut-off point of 9% for changes in coefficients or 1 for the odds ratio was used. The number of confounders identified was smaller than that identified with the significance method. The two methods were compared based on their performance using the likelihood ratio test, AIC, and BIC values. In this study, the significance testing approach outperforms the change in estimate. Maldonado and Greenland (38) stated that significance testing performed best when the significance value was set to 0.2. On the other hand, Talbot D et al. (49) questioned the ability of change in estimate to identify confounders due to its low ability to improve the precision of estimates.

It was found that mothers’ age at first birth, region, place of residence, mother education status, having radio and television, religion, household size, household wealth status, total children ever born, and birth order number were identified as confounders for the causal effect of a number of the antenatal care service on age-specific childhood vaccination. In addition, the number of antenatal care visits had a positive and significant effect on age-specific childhood vaccination, that is, when the number of antenatal care increased, the probability of getting age-specific childhood vaccination increased.

## 5 Conclusion

Zero-inflated Poisson regression best fits the relationship between pre-ANC covariates and ANC follow-up. The proportional odds model with a log link function also best fits pre-ANC covariates and age-specific childhood vaccination. Common cause, either treatment or outcome cause, and all pre-treatment covariate methods to select confounders did not show any significant variation. However, the common cause method provides a relatively smaller number of BIC values and a smaller number of covariates. Hence, the common cause method of confounder identification can be used if it performs better than other methods and provides a smaller number of covariates to control for when estimating the causal effect of an exposure on the outcome. The change in the estimate method of confounder selection provided a smaller number of confounders, and it is more conservative than significance testing when used at a 9% coefficient change and a *p*-value of 0.2, respectively. The likelihood ratio test demonstrates that the significance testing approach outperforms the change in estimate methods. Based on the findings of this study, it is important to control mothers’ age at first birth, region, place of residence, education status of mothers, presence of radio and television in the household, religion, household size, wealth status, total children ever born, and birth order number while estimating the causal effect of ANC on age-specific childhood vaccination. Increasing the number of antenatal care visits increases the likelihood of a child getting the required vaccines at each age interval.

## 6 Limitation

This study used data obtained from children who were alive at the time of the survey, which may introduce survivorship bias and could limit the generalizability of the study conclusion.

## Data Availability

We accessed the Ethiopian Demographic and Household Survey data from DHS online repository: (https://dhsprogram.com/data/available-datasets.cfm).
