# Correction: Identifying confounders and estimating the causal effect of antenatal care on age-specific childhood vaccination

**DOI:** 10.3389/fpubh.2026.1797800

**Published:** 2026-02-10

**Authors:** Ashagrie Sharew Iyassu, Haile Mekonnen Fenta, Zelalem G. Dessie, Temesgen T. Zewotir

**Affiliations:** 1 Department of Statistics, College of Science, Bahir Dar University, Bahir Dar, Ethiopia; 2 Department of Statistics, Debre Markos University, Debre Markos, Ethiopia; 3 Population Health, Center for Environmental and Respiratory Health Research, University of Oulu, Oulu, Finland; 4 School of Mathematics, Statistics and Computer Science, University of KwaZulu-Natal, Durban, South Africa

**Keywords:** antenatal care, childhood immunization, confounders, significance testing, change in estimate

Affiliation 1 “Department of Statistics, College of Science, Bahir Dar University, Bahir Dar, Ethiopia” was erroneously given as “College of Science, Bahir Dar University, Bahir Dar, Ethiopia”.

The original version of this article has been updated.

