# Supplementary material for: Identifying confounders and estimating the causal effect of antenatal care on age-specific childhood vaccination
Source: Front Public Health. 2025 May 30;13:1420567. doi: 10.3389/fpubh.2025.1420567 (PMC12162972; doi:10.3389/fpubh.2025.1420567)
Supplement: Supplementary file 1 [file Data_Sheet_1.pdf]

Supplementary material

Distribution of childhood vaccines missingness

Vaccines	Observed	% missing	observed	% missing	Vaccines	Observed	% observed	missing	% missing
BCG	3,103	59.0%	2,159	41.0%	Pneumococcal2	3,073	58.4%	2,189	41.6%
DPT 1	3,077	58.5%	2185	41.5%	Pneumococcal3	3,071	58.4%	2191	41.6%
POLIO 1	3,097	58.9%	2,165	41.1%	Rotavirus 1	3,062	58.2%	2200	41.8%
DPT 2	3,076	58.5%	2,186	41.5%	Rotavirus 2	3,062	58.2%	2200	41.8%
POLIO 2	3,097	58.9%	2,165	41.1%	Polio inactive	3,073	58.4%	2189	41.6%
DPT 3	3,075	58.4%	2,187	41.6%	Hepatitis B 1	3,077	58.5%	2185	41.5%
POLIO 3	3,096	58.8%	2,166	41.2%	Hepatitis B 2	3,076	58.5%	2186	41.5%
MEASLES 1	3,082	58.6%	2,180	41.4%	Hepatitis B 3	3,075	58.4%	2187	41.6%
MEASLES 2	3,076	58.5%	2,186	41.5%	HiB 1	3,077	58.5%	2185	41.5%
POLIO 0	3,089	58.7%	2,173	41.3%	HiB 2	3,076	58.5%	2186	41.5%
Pentavalent 1	3,077	58.5%	2,185	41.5%	HiB 3	3,075	58.4%	2187	41.6%
Pentavalent 2	3,076	58.5%	2,186	41.5%	Vitamin A	3,028	57.5%	2234	42.5%
Pentavalent 3	3,074	58.4%	2,188	41.6%					
Pneumococcal1	3,073	58.4%	2,189	41.6%	Over all vaccination	2,694	0.0%	2,568	48.8%

Test of missing at random (MAR) for childhood vaccination status

Observed covariate	UOR	Std. err.	P value	AOR	Std. err.	P value	[95% conf.	nterval]
ANC	0.992	0.015	0.584					
region								
Afar	1.075	0.136	0.566	0.906	0.167	0.593	0.631	1.301
Amhara	1.047	0.140	0.73	0.929	0.140	0.627	0.692	1.248
Oromia	1.029	0.127	0.819	0.939	0.153	0.697	0.682	1.291
Somali	1.332	0.170	**0.025**	1.118	0.211	0.554	0.772	1.618
Benishangul	0.995	0.133	0.969	0.942	0.157	0.722	0.679	1.307
SNNPR	1.269	0.159	0.058	1.145	0.191	0.418	0.826	1.587
Gambela	1.043	0.144	0.762	0.998	0.181	0.989	0.699	1.423
Harari	1.042	0.145	0.769	1.045	0.196	0.817	0.723	1.509
Addis Adaba	0.776	0.120	**0.102**	0.841	0.166	0.381	0.570	1.239
Dire Dawa	0.996	0.143	0.98	1.060	0.201	0.758	0.731	1.536
place_residence								
Rural	1.156	0.076	**0.027**	0.841	0.097	0.133	0.671	1.054
age_cat								

20-24	1.450	0.212	**0.011**	1.701	0.285	**0.002**	1.225	2.361
25-29	1.928	0.271	**0.000**	2.978	0.522	**0.000**	2.112	4.198
30-34	2.046	0.296	**0.000**	4.478	0.903	**0.000**	3.016	6.648
35-39	2.669	0.407	**0.000**	9.661	2.265	**0.000**	6.102	15.296
40-44	2.264	0.396	**0.000**	10.62	2.997	**0.000**	6.115	18.471
45-49	3.394	0.869	**0.000**	29.919	11.523	**0.000**	14.065	63.646
Education								
Primary	0.750	0.046	**0.000**	0.901	0.074	0.203	0.767	1.058
Secondary	0.688	0.070	**0.001**	0.972	0.130	0.831	0.748	1.263
Higher	0.666	0.084	**0.001**	0.945	0.159	0.738	0.680	1.314
radio								
Yes	1.070	0.068	0.288					
television								
Yes	0.778	0.056	**0.000**	0.942	0.145	0.701	0.697	1.275
religion								
Catholic	0.860	0.320	0.686	0.782	0.333	0.564	0.340	1.800
Protestant	1.100	0.091	0.247	1.068	0.132	0.594	0.838	1.360
Muslim	1.099	0.071	0.146	0.924	0.102	0.475	0.745	1.147
Traditional	1.452	0.355	0.128	1.075	0.319	0.807	0.601	1.924
hsize	1.052	0.012	**0.000**	0.953	0.018	**0.010**	0.919	0.989
sex_hh								
Female	0.981	0.067	0.775					
age_hh	1.009	0.003	**0.000**	1.008	0.004	**0.019**	1.001	1.015
wealth_index								
Poorer	0.899	0.074	0.194	1.047	0.109	0.658	0.854	1.284
Middle	0.891	0.078	0.188	1.085	0.120	0.461	0.873	1.349
Richer	0.955	0.086	0.606	1.175	0.138	0.167	0.934	1.479
Richest	0.721	0.055	**0.000**	1.047	0.184	0.794	0.742	1.476
Number of children ever born	1.100	0.013	**0.000**	7.842	0.610	**0.000**	6.733	9.133
age_1st_birth	0.980	0.007	**0.002**	0.902	0.010	**0.000**	0.882	0.922
marital_status								
Widowed	1.254	0.275	0.303	1.529	0.378	0.086	0.941	2.483
Divorced	1.242	0.167	**0.108**	1.714	0.261	**0.000**	1.272	2.309
child_sex								
Female	0.954	0.053	0.398					
place_delivery								
At health facility	0.704	0.039	**0.000**	0.833	0.064	**0.018**	0.716	0.970
child_twin								
twine	1.204	0.223	0.316					
BORD	1.015247	0.011714	**0.19**	0.103	0.008	**0.000**	0.089	0.121

Test of MAR for number of Antenatal care

ANC_missing	Unadjusted Odds ratio	z	P>z	Adjusted Odds ratio	z	P>z
region						
Afar	2.114	5.310	0.000	1.237	0.540	0.589
Amhara	0.840	-1.080	0.281	0.778	-0.640	0.522
Oromia	1.475	2.750	0.006	1.038	0.100	0.919
Somali	2.830	7.370	0.000	1.269	0.600	0.546
Benishangul	1.252	1.460	0.144	0.773	-0.660	0.510
SNNPR	1.355	2.110	0.035	1.271	0.630	0.530
Gambela	0.974	-0.160	0.870	0.548	-1.210	0.228
Harari	1.421	2.230	0.026	1.367	0.780	0.436
Addis Adaba	0.807	-1.140	0.256	0.858	-0.320	0.746
Dire Dawa	1.411	2.120	0.034	1.376	0.800	0.426
place_residence						
Rural	1.488	5.300	0.000	1.339	1.200	0.231
age_cat						
20-24	1.897	3.560	0.000	0.914	-0.280	0.782
25-29	2.447	5.170	0.000	0.395	-2.590	**0.010**
30-34	2.445	5.050	0.000	0.206	-3.640	**0.000**
35-39	1.780	3.090	0.002	0.079	-4.680	**0.000**
40-44	1.802	2.810	0.005	0.073	-4.160	**0.000**
45-49	1.410	1.130	0.257	0.008	-3.920	**0.000**
education						
Primary	0.753	-4.200	0.000	1.202	1.080	0.278
Secondary	0.543	-5.030	0.000	1.240	0.750	0.453
Higher	0.550	-3.980	0.000	1.449	1.050	0.295
television						
Yes	0.590	-6.240	0.000	1.328	0.860	0.390
religion						
Catholic	0.911	-0.200	0.841	0.952	-0.050	0.964
Protestant	1.237	2.200	0.028	0.705	-1.160	0.248
Muslim	2.105	9.980	0.000	1.251	0.900	0.367
Traditional	2.374	3.450	0.001	1.557	0.740	0.459
hsize	1.142	10.560	0.000	1.009	0.220	0.823
age_hh	0.992	-2.820	0.005	0.989	-1.420	0.155
wealth_index						
Poorer	0.687	-4.300	0.000	0.824	-0.890	0.371
Middle	0.557	-6.050	0.000	0.758	-1.090	0.277
Richer	0.573	-5.610	0.000	1.031	0.130	0.899
Richest	0.433	-9.660	0.000	0.735	-0.830	0.406

Children ever born	1.167	12.570	0.000	1.559	6.910	**0.000**
age_1st_birth	0.984	-2.260	0.024	1.148	5.290	**0.000**
marital_status						
Divorced	0.495	-4.060	0.000	1.325	0.780	0.434
vaccine_status	0.658	-3.480	0.000	0.876	-0.970	0.334

Sensitivity analysis of multiple imputation

	Exposure	Number of obs.	Coefficient	std.err	P value	95% CI		length of CI
Complete case analysis	ANC	3028	0.576	0.042	<0.01	0.494	0.658	0.164
Multiple imputation	ANC	5150	0.17	0.008	<0.01	0.154	0.186	0.032

Result of confounder identification analysis for exposure and outcome.

Analysis for outcome (age specific childhood vaccination)

```
Coefficients:
                    Estimate Std. Error z value Pr(>|z|)
as.factor(age_cat)2  0.39428    0.09045   4.359 1.31e-05 ***
as.factor(age_cat)3  0.43401    0.08680   5.000 5.73e-07 ***
as.factor(age_cat)4  0.45335    0.08937   5.073 3.92e-07 ***
as.factor(age_cat)5  0.51670    0.09437   5.475 4.37e-08 ***
as.factor(age_cat)6  0.53412    0.10881   4.909 9.17e-07 ***
as.factor(age_cat)7  0.91336    0.17056   5.355 8.55e-08 ***
as.factor(region)2  -1.02433    0.08936 -11.463  < 2e-16 ***
as.factor(region)3  -0.37546    0.09417  -3.987 6.69e-05 ***
as.factor(region)4  -0.61467    0.08730  -7.041 1.91e-12 ***
as.factor(region)5  -0.98750    0.09003 -10.968  < 2e-16 ***
as.factor(region)6  -0.45853    0.09351  -4.904 9.41e-07 ***
as.factor(region)7  -0.76289    0.08827  -8.642  < 2e-16 ***
as.factor(region)8  -0.58877    0.09610  -6.127 8.96e-10 ***
as.factor(region)9  -0.56238    0.09614  -5.850 4.93e-09 ***
as.factor(region)10  0.39656    0.14522   2.731  0.00632 **
as.factor(region)11 -0.03293    0.10694  -0.308  0.75811
as.factor(Place_residence)2 -0.59971    0.04536  -13.22   <2e-16 ***
as.factor(education)1      0.21256    0.03881   5.477 4.33e-08 ***
as.factor(education)2     0.45924    0.06741   6.813 9.59e-12 ***
as.factor(education)3     0.91801    0.10346   8.873  < 2e-16 ***
as.factor(radio)1  0.24911    0.04071    6.12 9.37e-10 ***
as.factor(television)1  0.61311    0.05059   12.12   <2e-16 ***
as.factor(religion)2  -0.35790    0.22839  -1.567    0.117
as.factor(religion)3  -0.39580    0.05276  -7.501 6.32e-14 ***
as.factor(religion)4  -0.44254    0.04202 -10.531  < 2e-16 ***
as.factor(religion)5  -0.98786    0.18639  -5.300 1.16e-07 ***
as.factor(religion)96  0.47904    0.43861   1.092    0.275
hsize              -0.020552   0.007563  -2.717  0.00658 **
as.factor(sex_hh)2 -0.03199    0.04309  -0.742    0.458
age_hh             0.003145     0.001543   2.038   0.0415 *
as.factor(wealth)2  0.33806    0.05117   6.607 3.93e-11 ***
as.factor(wealth)3  0.36414    0.05504   6.616 3.69e-11 ***
as.factor(wealth)4  0.56349    0.05825   9.674  < 2e-16 ***
as.factor(wealth)5  0.84420    0.05211  16.199  < 2e-16 ***
```

```
total_child        -0.017266   0.007147  -2.416   0.0157 *
age_1st            0.020278   0.004397   4.612    4e-06 ***
as.factor(marriage)1 -0.11811    0.24112  -0.490    0.624
as.factor(marriage)2 -0.06683    0.32348  -0.207    0.836
as.factor(marriage)3 -0.01913    0.29293  -0.065    0.948
as.factor(marriage)4 -0.07907    0.26209  -0.302    0.763
as.factor(marriage)5 -0.14119    0.28520  -0.495    0.621
BORD               -0.035756   0.007404  -4.829 1.37e-06 ***
```

Analysis on exposure (number of Antenatal care)

```
Count model coefficients (poisson with log link):
                     Estimate Std. Error z value Pr(>|z|)
as.factor(age_cat)2  0.085767   0.049054   1.748   0.0804 .
as.factor(age_cat)3  0.067201   0.047330   1.420   0.1557
as.factor(age_cat)4  0.081013   0.048655   1.665   0.0959 .
as.factor(age_cat)5  0.066075   0.051281   1.289   0.1976
as.factor(age_cat)6  0.020909   0.060305   0.347   0.7288
as.factor(age_cat)7 -0.009805   0.091584  -0.107   0.9147

as.factor(region)2  -0.33124    0.04146  -7.990 1.35e-15 ***
as.factor(region)3  -0.05205    0.03849  -1.352  0.17634
as.factor(region)4  -0.15149    0.03708  -4.086 4.39e-05 ***
as.factor(region)5  -0.64924    0.05387 -12.052  < 2e-16 ***
as.factor(region)6  -0.09755    0.03909  -2.495  0.01258 *
as.factor(region)7  -0.22448    0.03882  -5.783 7.34e-09 ***
as.factor(region)8  -0.29177    0.04453  -6.552 5.68e-11 ***
as.factor(region)9  -0.03953    0.03976  -0.994  0.32012
as.factor(region)10  0.19790    0.03927   5.040 4.66e-07 ***
as.factor(region)11  0.12207    0.03901   3.129  0.00175 **

as.factor(Place_residence)2 -0.36294    0.01868  -19.43   <2e-16 ***
as.factor(education)1  0.21750    0.02063   10.54   <2e-16 ***
as.factor(education)2  0.40209    0.02820   14.26   <2e-16 ***
as.factor(education)3  0.53299    0.03159   16.87   <2e-16 ***
as.factor(radio)1  0.17161    0.01927   8.908   <2e-16 ***
as.factor(television)1  0.40695    0.01932   21.07   <2e-16 ***
as.factor(religion)2  -0.39107    0.13674  -2.860  0.00424 **
as.factor(religion)3  -0.20112    0.02603  -7.726 1.11e-14 ***
as.factor(religion)4  -0.21752    0.01993 -10.915  < 2e-16 ***
as.factor(religion)5  -0.43326    0.13247  -3.271  0.00107 **
as.factor(religion)96 -0.55490    0.23428  -2.369  0.01786 *
hsize       -0.045520   0.004098  -11.11   <2e-16 ***
as.factor(sex_hh)2  0.01556    0.02261   0.688    0.491
age_hh            0.0005731  0.0008421   0.681    0.496
as.factor(wealth)2  0.22697    0.03133   7.244 4.36e-13 ***
as.factor(wealth)3  0.30772    0.03207   9.595  < 2e-16 ***
as.factor(wealth)4  0.38644    0.03127  12.357  < 2e-16 ***
as.factor(wealth)5  0.60813    0.02597  23.417  < 2e-16 ***
total_child -0.055940   0.004077  -13.72   <2e-16 ***
age_1st     0.019975   0.002034   9.818   <2e-16 ***
as.factor(marriage)1 -0.074042   0.129070  -0.574    0.566
as.factor(marriage)2  0.103350   0.169019   0.611    0.541
as.factor(marriage)3 -0.241230   0.159053  -1.517    0.129
as.factor(marriage)4 -0.007964   0.138535  -0.057    0.954
as.factor(marriage)5  0.051969   0.148561   0.350    0.726
BORD        -0.037427   0.004144  -9.031   <2e-16 ***
```

Result for the effect of ANC on age specific childhood vaccination when identified confounders are adjusted with regression

```
Coefficients:
                            Estimate Std. Error z value Pr(>|z|)    
ANC_num                     0.101266   0.010837   9.345  < 2e-16 ***
age_1st                     0.008008   0.005445   1.471 0.141388    
as.factor(region)2         -0.960859   0.123887  -7.756 8.77e-15 ***
as.factor(region)3         -0.347706   0.104383  -3.331 0.000865 ***
as.factor(region)4         -0.583972   0.112724  -5.181 2.21e-07 ***
as.factor(region)5         -0.910984   0.125245  -7.274 3.50e-13 ***
as.factor(region)6         -0.396330   0.114151  -3.472 0.000517 ***
as.factor(region)7         -0.696153   0.113701  -6.123 9.20e-10 ***
as.factor(region)8         -0.433633   0.121753  -3.562 0.000369 ***
as.factor(region)9         -0.798346   0.130460  -6.119 9.39e-10 ***
as.factor(region)10        -0.170638   0.173159  -0.985 0.324406    
as.factor(region)11        -0.196039   0.138335  -1.417 0.156445    
as.factor(Place_residence)2 -0.347823   0.071917  -4.836 1.32e-06 ***
as.factor(education)1       0.033677   0.049487   0.681 0.496174    
as.factor(education)2      -0.024319   0.088111  -0.276 0.782544    
as.factor(education)3       0.327929   0.132773   2.470 0.013517 *  
radio                       0.045937   0.051122   0.899 0.368874    
television                 -0.057856   0.099041  -0.584 0.559109    
as.factor(religion)2       -0.031782   0.252101  -0.126 0.899677    
as.factor(religion)3       -0.111995   0.077176  -1.451 0.146734    
as.factor(religion)4        0.009816   0.073691   0.133 0.894027    
as.factor(religion)5       -0.520650   0.202217  -2.575 0.010032 *  
as.factor(religion)96       0.886413   0.496046   1.787 0.073944 .  
hsize                      -0.016447   0.011536  -1.426 0.153966    
as.factor(wealth)2          0.146518   0.060747   2.412 0.015868 *  
as.factor(wealth)3          0.189397   0.067303   2.814 0.004892 ** 
as.factor(wealth)4          0.360510   0.074449   4.842 1.28e-06 ***
as.factor(wealth)5          0.307084   0.107143   2.866 0.004156 ** 
total_child                 0.539082   0.038160  14.127  < 2e-16 ***
BORD                       -0.520590   0.038155 -13.644  < 2e-16 ***
---
Signif. codes:  0 ‘***’ 0.001 ‘**’ 0.01 ‘*’ 0.05 ‘.’ 0.1 ‘ ’ 1

Threshold coefficients:
    Estimate Std. Error z value
0|1  -0.8447     0.1760    -4.8
1|2   3.5521     0.1909    18.6
```
